# Exploring transdiagnostic factors of internalizing and externalizing symptoms in transitional-age youth: Using a variable-centered and person-centered approach

**DOI:** 10.1007/s12144-025-08328-3

**Published:** 2025-09-04

**Authors:** Janna Keulen, Maja Deković, Leentje Vervoort, Denise Bodden

**Affiliations:** 1 Department of Clinical Child & Family Studies, Utrecht University, Utrecht, The Netherlands; 2 Behavioural Science Institute, Radboud Universiteit Nijmegen, Nijmegen, The Netherlands

**Keywords:** Transitional-age youth, Transdiagnostic factors, Internalizing symptoms, Externalizing symptoms, CIBER, Latent profile analysis

## Abstract

Transitional-age youth (TAY; 15 to 25 years old) are more likely to experience psychological problems compared to other age groups. This study aimed to identify the most relevant transdiagnostic factors underlying internalizing and externalizing symptoms in TAY, including perfectionism, perceived stress, self-compassion, psychological flexibility, adaptive and maladaptive emotion regulation, self-esteem, and autonomy. The sample consisted of 87 TAY from a clinical and 649 from a community sample (M = 20.71, 64.1% female). Confidence Interval Based Estimation of Relevance (CIBER) was used to detect the most relevant transdiagnostic factors, and Latent Profile Analyses (LPA) were used to identify groups of TAY sharing similar patterns of transdiagnostic factors. CIBER showed that all transdiagnostic factors were associated with both internalizing and externalizing symptoms, with most factors being more strongly associated with internalizing symptoms. LPA identified six groups of TAY: low resilience (6.5%), moderate low resilience (20.5%), average resilience (29.3%), moderate high resilience (26.8%), moderate high resilience – high perfectionism and autonomy (1.8%) and high resilience (15.1%). Generally, TAY in the lower resilience groups were more likely to be in the clinical sample than in the community sample and showed more symptoms compared to TAY in the higher resilience groups. Conversely, TAY in the moderate high resilience – high perfectionism and autonomy group were most likely to be in the community sample. The study highlights the importance of focusing on transdiagnostic factors in research and clinical practice for TAY

The transitional period from middle adolescence (starting at 15-years-old) to early adulthood (until 25-years-old) is a critical and challenging period characterized by many social, cognitive and environmental transitions (e.g., becoming self-reliant, exploring sexuality, forming an identity, finishing education and choosing a career) (Martel & Fuchs, 2017; Wilens & Rosenbaum, 2013). Youth in this age group, called transitional age youth (TAY) (Wilens & Rosenbaum, 2013), are more likely to experience psychological problems compared to other age groups (Whiteford et al., 2013). If left untreated, these psychological problems can persist into adulthood, turn into severe or chronic disorders and/or are associated with comorbid problems (Costello & Maughan, 2015; Gustavson et al., 2018). Also, TAY with psychological problems often experience a lower quality of life and engage less in meaningful activities, such as education, work and social events compared to TAY without psychological problems (Patel et al., 2007). Therefore, gaining a better understanding of the most relevant factors related to the development and maintenance of psychological problems in this specific age group is essential, as this knowledge can improve prevention and intervention programs for TAY.

Traditionally, research on psychopathology and intervention programs often focus on specific psychological disorders, classified by the Diagnostic and Statistical Manual of Mental Disorders, Fifth Edition (DSM-5) (American Psychiatric Association, 2013) or International Classification of Diseases, Tenth Edition (ICD-10) (World Health Organization, 2019). While these frameworks provide structure for understanding and treating psychological problems, they may not fully address the needs of TAY, who often experience comorbid problems, have symptom profiles that change over time or do not show enough symptoms or the right combination of symptoms to receive a DSM-5 or ICD-10 diagnosis (Kessler et al., 2005; Marchette & Weisz, 2017). Hence, examining transdiagnostic factors that underlie the symptoms of multiple disorders provides a broader perspective, potentially better suited to the complex needs of TAY. Transdiagnostic factors can refer to biological (e.g., executive functioning deficits), socio-environmental (e.g., stressful life events) and psychological (e.g., emotion regulation) factors (Lynch et al., 2021). In this study, we solely focused on psychological transdiagnostic factors as these factors are most amenable to psychological prevention and intervention methods.

Specifically, we explored *perfectionism* (i.e., setting excessively high standards for performance accompanied by overly critical self-evaluation) (Frost et al., 1990), *perceived stress* (i.e., the degree to which situations are appraised as stressful) (Cohen et al., 1983), *self-compassion* (i.e., treating oneself with kindness, recognizing one’s shared humanity, and being mindful when considering negative aspects of oneself) (Neff, 2003a), *psychological flexibility* (i.e., the ability to contact the present moment more fully as a conscious human being, and to change or persist in behavior when doing so serves valued ends) (Hayes et al., 2006), *adaptive and maladaptive emotion regulation* (i.e., extrinsic and intrinsic processes responsible for monitoring, evaluating, and modifying emotional reactions to accomplish one’s goals, encompassing both adaptive and maladaptive strategies) (Thompson, 1994), *self-esteem* (i.e., evaluative aspect of self-knowledge that concerns the extent to which people like themselves) (Brown & Marshall, 2006) and *autonomy* (i.e., one’s ability to choose and define goals, feel confident about one’s own choices and goals and develop a strategy to achieve one’s goals) (Noom et al., 2001).

We selected these transdiagnostic factors as several studies, including meta-analyses, show that individuals with higher levels of perfectionism (Limburg et al., 2017), perceived stress (Felton et al., 2017; Thorsen et al., 2016) and maladaptive emotion regulation (Aldao et al., 2010), and lower levels of self-compassion (MacBeth & Gumley, 2012; Zessin et al., 2015), psychological flexibility (Doorley et al., 2020), adaptive emotion regulation (Aldao et al., 2010), self-esteem (Zeigler-Hill, 2011) and autonomy (Ryan et al., 2016) are more vulnerable to experience psychological problems than individuals with lower levels of perfectionism, perceived stress and maladaptive emotion regulation and higher levels of self-compassion, psychological flexibility, adaptive emotion regulation, self-esteem and autonomy. Moreover, these factors are all psychological transdiagnostic factors that are already often targeted in existing transdiagnostic interventions, such as Compassion Focused Therapy (targeting self-compassion) (Craig et al., 2020), Acceptance and Commitment Therapy (targeting psychological flexibility) (Hayes et al., 2006), Competitive Memory Training (targeting self-esteem) (Korrelboom et al., 2022), and the Unified Protocol (targeting emotion dysregulation) (Sakiris & Berle, 2019). Notwithstanding, it is still unclear which of these transdiagnostic factors are the most relevant psychological transdiagnostic factors for internalizing and externalizing symptoms in TAY, as no study that we are aware of has examined these factors simultaneously.

Therefore, the first aim of this study was to identify which of these proposed transdiagnostic factors are most relevant for internalizing and externalizing symptoms in TAY. To evaluate this, we examined two important things. First, we explored the distribution of each transdiagnostic factor across TAY (Crutzen et al., 2017; Peters & Crutzen, 2018). Particularly, we investigated which transdiagnostic factors most TAY experience difficulties with (i.e., high levels of perfectionism, stress, maladaptive emotion regulation and/or low levels of self-compassion, psychological flexibility, self-esteem, and autonomy). If a large proportion of TAY experience difficulties with a particular transdiagnostic factor, that factor may be a more relevant target for prevention and intervention programs, as there is generally greater potential for improvement compared to transdiagnostic factors on which most TAY do not experience difficulties (Crutzen et al., 2017; Peters & Crutzen, 2018). Second, we investigated the strength of the association between each transdiagnostic factor, on one hand and internalizing and externalizing symptoms, on the other hand. Specifically, the stronger a transdiagnostic factor is related to internalizing and externalizing symptoms, the more relevant it is to target that factor in prevention and intervention programs for TAY. Thus, the transdiagnostic factors that have the most potential for improvement and the strongest association with internalizing and externalizing symptoms are possible the most relevant psychological transdiagnostic factors to target in preventions and interventions.

To identify the most relevant transdiagnostic factors, we applied Confidence Interval Based Estimation of Relevance (CIBER) approach. In CIBER, sample means (as an index of the sample distribution of the transdiagnostic factors) and the correlation coefficients (as an index of the association between the transdiagnostic factors and internalizing and externalizing symptoms) and their respective confidence intervals are visually presented. The advantage of CIBER, compared to traditional regression approaches, is that it does not remove the overlap between predictors in their explanation of the outcome. This allows to identify the univariate association between each predictor variable (i.e., one transdiagnostic mechanism) with the outcome (i.e., internalizing and externalizing symptoms) (Crutzen et al., 2017; Peters & Crutzen, 2018). CIBER is a relative new approach, but there are already some studies that used this variable-centered approach to establish the most relevant determinants for 3,4-Methyl​enedioxy​methamphetamine (MDMA) use in young adults (Crutzen et al., 2017; Peters & Crutzen, 2018) and problematic eating behavior in children and adolescents (Vervoort et al., 2020). As, to our knowledge, the current study was the first study using the CIBER approach to establish the most relevant transdiagnostic factors of internalizing and externalizing symptoms in TAY, no hypotheses were formulated.

Although the literature shows that the abovementioned transdiagnostic factors (e.g., perfectionism, self-compassion, etc.) are related to psychological symptoms in TAY, these factors have typically been examined separately. This is not optimal, as transdiagnostic factors often co-occur. For instance, Rudolph et al. (2007) found that young adults with higher levels of perfectionistic cognitions were also more likely to use maladaptive emotion regulation strategies (e.g., catastrophizing, self-blame, rumination, and lack of positive reappraisal) compared to their less perfectionistic peers. Furthermore, individuals with higher levels of self-compassion were also found to have higher levels of psychological flexibility than individuals with lower levels of self-compassion (Marshall & Brockman, 2016). The co-occurrence of several transdiagnostic factors may also decrease or increase TAY’s risk of experiencing psychological symptoms. For instance, adolescents and adults with higher levels of perfectionism were found to be more vulnerable to experience depressive symptoms compared to adolescents and adults with lower levels of perfectionism. However, higher levels of self-compassion buffered the effects of maladaptive perfectionism on depression (Ferrari et al., 2018). Hence, there may be relevant heterogeneity in patterns of transdiagnostic factors within TAY, and these patterns may relate differently to internalizing and externalizing symptoms. This heterogeneity cannot be detected with variable-centered approaches in which only associations between transdiagnostic factors and internalizing and externalizing symptoms are studied. To detect this heterogeneity, person-centered approaches, such as Latent Profile Analyses (LPA) (Williams & Kibowski, 2016) are needed.

Therefore, to complement to a variable-centered approach (i.e., CIBER), the second aim of this study was to identify groups of TAY sharing similar patterns of transdiagnostic factors, using a person-centered approach (i.e., LPA). After identifying the groups, we investigated if the groups differed on three mental health related outcomes. Specifically, we investigated if the groups differed in their overall levels of internalizing symptoms and externalizing symptoms and if TAY who were recruited via health care organizations (i.e., clinically referred TAY meeting the criteria of a DSM-5 diagnosis) were more likely to be in certain groups compared TAY who were recruited from the general population, and vice versa. Studying this heterogeneity is clinically relevant as it potentially aids to the development of tailored psychological prevention and intervention methods for specific groups of TAY (Nye et al., 2023).

Earlier studies that explored the heterogeneity of transdiagnostic factors in adolescents and/or young adults often focused solely on one transdiagnostic factor, such as perfectionism (Ruiz-Esteban et al., 2021; Stornæs et al., 2019), self-compassion (Chi et al., 2022; Ferrari et al., 2022), psychological flexibility (Bi & Li, 2021) and emotion regulation (Dixon-Gordon et al., 2015; McGlinchey et al., 2021; Van Den Heuvel et al., 2020). Some of these studies identified groups mainly differing in their general levels of these transdiagnostic factors (e.g., high, moderate and low psychological flexibility and emotion regulation) (Bi & Li, 2021; McGlinchey et al., 2021). Notwithstanding, other studies also labeled groups as more adaptive or maladaptive (e.g., adaptive and maladaptive perfectionists and emotion regulators) (Dixon-Gordon et al., 2015; Ruiz-Esteban et al., 2021; Stornæs et al., 2019; Van Den Heuvel et al., 2020). Individuals in the adaptive groups showed less psychological symptoms and higher resilience compared to individuals in the maladaptive groups.

Considering these earlier LPA studies, we expected to find groups that differ in their general levels of several transdiagnostic factors, but also could be labeled as more or less resilient based on their associations with the three mental health related outcome (i.e., internalizing symptoms, externalizing symptoms and type of sample [clinical or community sample]). Specifically, given the vulnerability of the transitional age period, we expected that TAY with high perfectionism, stress, and maladaptive emotion regulation, along with low self-compassion, psychological flexibility, self-esteem, and autonomy, would form a low resilient group with relatively high levels of internalizing and externalizing symptoms. Conversely, TAY with the opposite characteristics were anticipated to form a high resilient group, exhibiting relatively low levels of psychological symptoms. TAY with moderate levels across all transdiagnostic factors were expected to represent a moderate resilient group, displaying moderate levels of internalizing and externalizing symptoms. Additionally, we considered the possibility of identifying groups of TAY that predominantly encounter challenges in specific areas. For example, one group of TAY might struggle primarily with self-related issues (e.g., low self-compassion and self-esteem), while another group might predominantly encounter difficulties in taking purposeful steps toward values and goals, exhibiting low psychological flexibility and autonomy. However, as, to our knowledge this is the first study performing an LPA on various transdiagnostic factors, no specific hypotheses were specified.

## Method

### Participants

The study used data from two samples. A first sample of 87 TAY was recruited via several outpatient clinics in the Netherlands (i.e., clinical sample). The data was collected as part of a randomized controlled trial (RCT) studying the (cost-)effectiveness of Acceptance and Commitment Therapy (ACT) for TAY (Keulen et al., 2023). In the current study, only the baseline data was used. TAY from the clinical sample met the following inclusion criteria: (1) meeting the criteria of a DSM-5 disorder (except substance abuse, psychotic and/or bipolar disorder), (2) being 14 to 25 years old, 3) having an estimated IQ above 80, (4) having no acute suicide risk, (4) not currently meeting the criteria for a DSM-5 substance abuse, psychotic and/or bipolar disorder, (6) having sufficient knowledge of the Dutch language. A second sample of 649 TAY was recruited from the general population (i.e., community sample). TAY from the community sample met the following inclusion criteria: (1) being 15 to 25 years old and (2) having sufficient knowledge of the Dutch language.

In total, 736 TAY aged 14 to 26 (*M* = 20.71, *SD* = 3.02) participated in this study. There were 457 (62.1%) females, 268 (36.4%) males and 11 (1.5%) TAY identified themselves as non-binary. In total, 313 TAY (42.5%) were currently attending higher education, 211 TAY (28.7%) middle education and 13 TAY (1.8%) lower education. For 7 TAY (1.0%) the current education was unclear or missing. In addition, 196 TAY (26.6%) finished higher education, 336 TAY (45.7%) middle education and 195 TAY (26.5%) lower education. For 9 (1.2%) TAY the finished education was unclear or missing. Moreover, 698 (94.8%) TAY were born in the Netherlands, 653 TAY (88.7%) had a mother who was born in the Netherlands and 651 TAY (88.5%) had a father who was born in the Netherlands.

The two samples differed in their demographics. Specifically, TAY in the clinical sample were significantly younger (*M* = 17.63, *SD* = 2.22) compared to TAY in the community sample (*M* = 21.12, *SD* = 2.88; *t*(128.4)=−13.27, *p* <.001) and there were relatively more TAY who identified themselves as female or non-binary in the clinical sample (20.7% male, 72.4% female and 6.9% non-binary) than in the community sample (38.5% male, 60.7% female and 0.8% non-binary; *Χ²*(2) *=* 27.66, *p* <.001). In addition, TAY in the clinical sample were relatively more likely to currently attend (*Χ²*(3) *=* 74.74 *p* <.001) and/or finished (*Χ²*(3) *=* 79.84, *p* <.001) low or middle education (current education: 8.0% low, 62.1% middle and 11.5% high; highest finished education: 64.4% low, 33.3% middle and 2.3% high) compared to TAY in the community sample (current education: 0.9% low, 24.2% middle and 46.7% high; highest finished education: 21.4% low, 47.3% middle and 29.9% high). No significant differences were found for the country of birth of TAY (*Χ²*(5) *=* 8.32, *p* < = 0.140), their mothers (*Χ²*(7) *=* 2.98, *p* =.887) and/or their fathers (*Χ²*(8) *=* 13.71, *p* =.090).

### Procedure

This study employed a cross-sectional design where data were collected at one single time point. The study of the clinical sample was approved by the Medical Research Ethics Committee NedMec (NL78679.041.21) and the study of the community sample was approved by the Ethics Review Board of Utrecht University's Faculty of Social and Behavioral Sciences (22-0049). TAY from the clinical sample were recruited by therapists and researchers working at the participating mental health care institutions and TAY from the community sample were recruited by bachelor and master students, using convenience sampling (e.g., via their own network, at educational institutions or in the local neighborhood of the recruiting students). Before participation, all TAY and parents/caregivers of TAY under the age of 16 provided written consent. After giving consent, TAY received a link for an online questionnaire. TAY could choose to fill out the questionnaire by themselves at home or together with a research assistant (clinical sample) or student (community sample). After filling out the questionnaire, TAY from the clinical sample received a 5 euro gift card and TAY from the community sample received a chocolate bar as a reward.

### Measures

Perfectionism was assessed using the Frost Multidimensional Perfectionism Scale (FMPS) (Frost et al., 1990), which includes 35 questions rated on a 5-point Likert scale ranging from 1=“*strongly disagree*” to 5=“*strongly agree*”. For the present study, we focused solely on the following subscales of the FMPS: personal standards, organization, concern over mistakes, and doubt about actions, totaling 26 questions. The subscales related to parent expectations and parental criticism were excluded to reduce time and effort for TAY. The reliability and validity of the FMPS were already established in previous studies (Frost et al., 1990). The Cronbach’s alpha for the FMPS subscales in our study was 0.91, indicating excellent internal consistency (Cronbach, 1951).

Perceived stress was measured using the Perceived Stress Scale (PSS-10) (Cohen et al., 1983; Van der Ploeg & Van der Ploeg, 2013). The PSS-10 consists of 10 questions rated on a 5-point Likert scale, ranging from 1=“*never*” to 5=“*very often*”. Previous research has shown acceptable psychometric properties for the PSS-10 (Lee, 2012). The Cronbach’s alpha for the PSS-10 in our study was 0.82, suggesting good internal consistency (Cronbach, 1951).

Self-compassion was evaluated with the Self-Compassion Scale Short Form (SCS-SF) (Neff, 2003b, 2016; Raes et al., 2011). The questionnaire consists of 12 items answered on a 7-point Likert scale, ranging from 1="*seldom or never*” to 7="*almost always*”. Previous research has confirmed the scale’s psychometric properties (Neff, 2003b, 2016; Raes et al., 2011). In our study, the Cronbach’s alpha for the SCS-SF was 0.87, demonstrating good internal consistency (Cronbach, 1951).

Psychological flexibility was measured using the Acceptance and Fusion Questionnaire for Youth (AFQ-Y) (Greco et al., 2008; Simon & Verboon, 2016). The AFQ-Y consists of 17 items rated on a 5-point Likert scale, ranging from 1="*not at all true*” to 5="*very true*”. Previous research has shown the AFQ-Y to have strong psychometric properties in both adolescent and adult samples (Fergus et al., 2012; Greco et al., 2008). In our study, the Cronbach’s alpha for the AFQ-Y was 0.90, representing excellent internal consistency (Cronbach, 1951).

Emotion regulation was assessed using the FEEL-KJ (Beveren et al., 2020), which measures a wide range of emotion regulation strategies that are used in response to anger, sadness, and anxiety. The FEEL-KJ distinguishes between maladaptive emotion regulation strategies (e.g., withdrawal, self-devaluation, rumination) and adaptive emotion regulation strategies (e.g., problem solving, distraction, acceptance). The questionnaire consists of 90 questions (30 questions for each emotion) rated on a 5-point Likert scale, ranging from 1="*almost never*” to 5="*almost always*”. To reduce burden for participants, TAY were asked to answer the 30 questions only for their most extreme emotion, choosing either anger, sadness, or anxiety. The FEEL-KJ has been found to have adequate psychometric properties (Cracco et al., 2015). For this study, the Cronbach’s alpha for the maladaptive subscale was 0.78, and the Cronbach’s alpha for the adaptive subscale was 0.84, showing good internal consistency (Cronbach, 1951).

Self-esteem was evaluated using the Rosenberg Self-Esteem Scale (RSES) (Franck et al., 2008; Rosenberg, 1965). This scale comprises 10 questions rated on a 4-point Likert scale, ranging from 1="*totally agree*” to 4="*totally disagree*”. Previous research has confirmed the validity and test-retest reliability of the Dutch version of the RSES (Franck et al., 2008). In our study, the Cronbach’s alpha for the RSES was 0.91, indicating excellent internal consistency (Cronbach, 1951).

Autonomy was measured with the Autonomy Adolescent Questionnaire (AAQ) (Noom et al., 2001). The AAQ categorizes autonomy into three main dimensions: attitudinal, emotional, and functional autonomy. The AAQ consists of 18 items rated on a 5-point Likert scale, ranging from 1="*not at all true for me*” to 5="*totally true for me*”. Previous research has provided support for the factor structure, convergent validity, and divergent validity of the AAQ (Noom et al., 2001). In our study, the Cronbach’s alpha for the AAQ was 0.88, suggesting good internal consistency (Cronbach, 1951).

Internalizing and externalizing symptoms were assessed with the Youth Self Report (YSR) (Verhulst et al., 1997). Some questions were slightly adjusted to make them more developmentally sensitive for young adults above the age of 18 (e.g., “I cut classes or skip school” was adjusted to “I cut classes or skip school/work”). To reduce time and effort for TAY, we only included the subscales measuring internalizing (anxious/depressed + withdrawn/depressed + somatic complaints) and externalizing problems (rule-breaking behaviour + aggressive behaviour). The YSR contains 69 items that are rated on a 3-point Likert scale ranging from 1=“*not true*” to 3=“*very true or often true*”. The psychometric properties of the YSR are adequate (Verhulst et al., 1997). In our study, the Cronbach’s alpha for the internalizing subscale was 0.93 and for the externalizing subscale was 0.86, demonstrating excellent and good internal consistency (Cronbach, 1951).

For all measures, items were recoded and mean scores were calculated, so that higher mean scores represented higher levels of perfectionism, stress, self-compassion, psychological flexibility, adaptive and maladaptive emotion regulation, self-esteem, autonomy and internalizing and externalizing symptoms.

### Data analysis

#### CIBER analysis

To examine which transdiagnostic factors (i.e., perfectionism, perceived stress, self-compassion, psychological flexibility, emotion regulation, self-esteem and autonomy) are the most relevant transdiagnostic factors related to internalizing and externalizing symptoms in TAY, a CIBER analysis was performed. To make the means comparable, we rescaled all eight transdiagnostic factors to be on the same scale ranging from 1 to 5. We used the procedure described on the IBM website (IBM, 2020). The outcomes (i.e., internalizing and externalizing symptoms) were not rescaled and ranged from 1 to 3. For the CIBER analysis, we used the free R package “userfriendlyscience” (Peters, 2017). The CIBER plot’s title presents the confidence interval of the proportion of variance in internalizing and externalizing symptoms that can be explained by all the transdiagnostic factors together. The names of the eight transdiagnostic factors are shown to the left of the left-hand panel. The CIBER output contains two panels. In the left-hand panel, for each transdiagnostic factor, the means are presented by diamonds showing the point estimates and the 99.99% confidence intervals of the means (i.e., representing the distribution of the transdiagnostic factors across TAY). The color of the diamond is indicative of the mean. Red diamonds represent lower means and green diamonds represent higher means, with more intense shades representing more extreme scores. Blue diamond represent means around the middle of the scale. The individual scores of the participants on each transdiagnostic factor are presented by the grey dots surrounding the diamonds. In the right-hand panel, the correlation coefficients between each transdiagnostic factor with internalizing and externalizing symptoms are presented by diamonds showings the point estimates and the 95% confidence intervals of the correlation coefficients (i.e., representing the strength of the associations between the transdiagnostic factors with internalizing and externalizing symptoms). The color of the diamond is indicative of the strength and direction of the correlation. Green diamonds represent positive correlations and red diamonds represent negative correlation, with more intense shades representing stronger correlations. Grey diamonds represent weak correlations, with more intense shades representing weaker correlations.

#### LPA analysis

To identify groups of TAY sharing similar patterns of transdiagnostic factors, an LPA was applied to the mean scores of the eight transdiagnostic factors (i.e., perfectionism, perceived stress, self-compassion, psychological flexibility, emotion regulation, self-esteem and autonomy) using Mplus version 8.3 (Muthén & Muthén, 2017). We compared the most parsimonious one-profile model to successive models with increasing numbers of profiles. To choose the most optimal profile solution we inspected several fit criteria. Specifically, lower values of the Bayesian Information Criterion (BIC) (Bollen & Long, 1993), the Sample Adjusted Bayesian Information Criterion (SA-BIC), and the Akaike’s Information Criterion (AIC), indicated better model fit. Moreover, a significant Lo-Mendell-Rubin adjusted likelihood ratio test (LMR) (Lo et al., 2001) and bootstrap likelihood ratio test (BLRt) (Langeheine et al., 1996) indicated a better model fit of the model under investigation compared to the model with one profile less. Additionally, entropy *R*^*2*^ values approaching one, indicated better model fit (Celeux & Soromenho, 1996). Last, mean class probabilities larger than 0.80 indicated sufficient model fit. In the current study, the BIC and BLRt values were prioritized as they are considered the most robust parameters of LPA model fit (Nylund et al., 2007). Additionally, models with small profile sizes (i.e., less than 5% of the sample size) and non-meaningful patterns were avoided (Collins & Lanza, 2010).

Next, to examine if groups differed in their overall levels of internalizing and externalizing symptoms and if TAY from the clinical sample were more likely to be in certain groups compared TAY who from the community sample, and vice versa, a multinomial logistic regression was performed in Mplus (Muthén & Muthén, 2017). In three separate analyses, the profiles were regressed on internalizing symptoms, externalizing symptoms and type of sample (dummy coded), using the 3-step approach. In a 3-step approach, the predictors are added to the model after the latent profiles are estimated and most likely profile membership is determined. This approach is optimal as it considers the degree of classification uncertainty (Asparouhov & Muthén, 2014; Vermunt, 2010).

#### Missing data

Item-level nonresponse was addressed through mean imputation in SPSS. In the LPA, missing data from construct-level nonresponse was imputed using full information maximum likelihood estimation in Mplus version 8.3 (Muthén & Muthén, 2017).

## Results

### Descriptive and missing analyses

Descriptive statistics and intercorrelations of the transdiagnostic factors and internalizing and externalizing symptoms are presented in Table 1. The intercorrelations show that all eight transdiagnostic factors were moderately to strongly related to each other (Cohen, 1988). Hence, although not included in the data analysis plan, to explore how these eight transdiagnostic factors group together, an exploratory factor analysis was conducted (see Appendix). The two samples differed in their scores on the transdiagnostic factors and internalizing and externalizing symptoms. Specifically, TAY in the clinical sample had higher scores on perfectionism (*t*(731) =−4.77, *p* <.001), stress (*t*(731)=−8.65, *p* <.001), maladaptive emotion regulation (*t*(732)=−9.51, *p* <.001), internalizing symptoms (*t*(734)=−12.80, *p* <.001) and externalizing symptoms (*t*(734)=−4.80, *p* <.001) and lower scores on self-compassion (*t*(731) = 10.12, *p* <.001), psychological flexibility (*t*(733) = 9.90, *p* <.001), adaptive emotion regulation (*t*(732) = 5.73, *p* <.001), self-esteem (*t*(731) = 12.35, *p* <.001) and autonomy (*t*(732) = 8.41, *p* <.001) compared to TAY in the community sample^1^. There were three TAY who did not finish the questionnaire and had missing scores on at least four of the seven transdiagnostic factors. These TAY did not differ from the other TAY in age (*t*(734) =−0.22, *p* =.415), gender (*χ*^2^(2) = 0.1, *p* =.969), internalizing symptoms (*t*(734) = 1.56, *p* =.119) and externalizing symptoms (*t*(734) = 0.27, *p* =.789). Moreover, these TAY were not more likely to be in either the clinical or community sample (*χ*^2^(1) = 1.34, *p* =.247).

**Table 1:** Descriptive statistics and intercorrelations of the transdiagnostic factors and internalizing and externalizing symptoms

	Total		Clinical Sample		Community Sample										
	M	SD	M	SD	M	SD	1.	2.	3.	4.	5.	6.	7.	8.	9.
1. Perfectionism	2.90	0.64	3.21	0.68	2.86	0.63									
2. Perceived stress	2.78	0.64	3.32	0.57	2.71	0.61	0.42								
3. Self-compassion	3.21	0.70	2.54	0.65	3.30	0.66	− 0.56	− 0.70							
4. Psychological flexibility	3.92	0.71	3.19	0.75	4.02	0.64	− 0.52	− 0.61	0.71						
5. Adaptive ER	3.36	0.60	2.98	0.65	3.41	0.58	− 0.12	− 0.37	0.42	0.29					
6. Maladaptive ER	2.65	0.67	3.26	0.61	2.57	0.64	0.42	0.59	− 0.64	− 0.67	− 0.30				
7. Self-esteem	3.69	0.86	2.71	0.80	3.83	0.79	− 0.49	− 0.68	0.80	0.70	0.39	− 0.62			
8. Autonomy	3.31	0.59	2.82	0.53	3.37	0.57	− 0.35	− 0.61	0.65	0.58	0.33	− 0.53	0.70		
9. Internalizing symptoms	1.43	0.34	1.83	0.32	1.38	0.31	0.43	0.63	− 0.64	− 0.76	− 0.30	0.62	− 0.71	− 0.60	
10. Externalizing symptoms	1.23	0.20	1.32	0.25	1.22	0.19	0.10	0.32	− 0.36	− 0.42	− 0.22	0.41	− 0.36	− 0.21	0.48

All correlations were significant at the 0.01 level. ER = Emotion Regulation. The scales of variables 1–8 range from 1–5 and the scales from variables 9–10 range from 1–3

### CIBER analysis

**Fig. 1:**
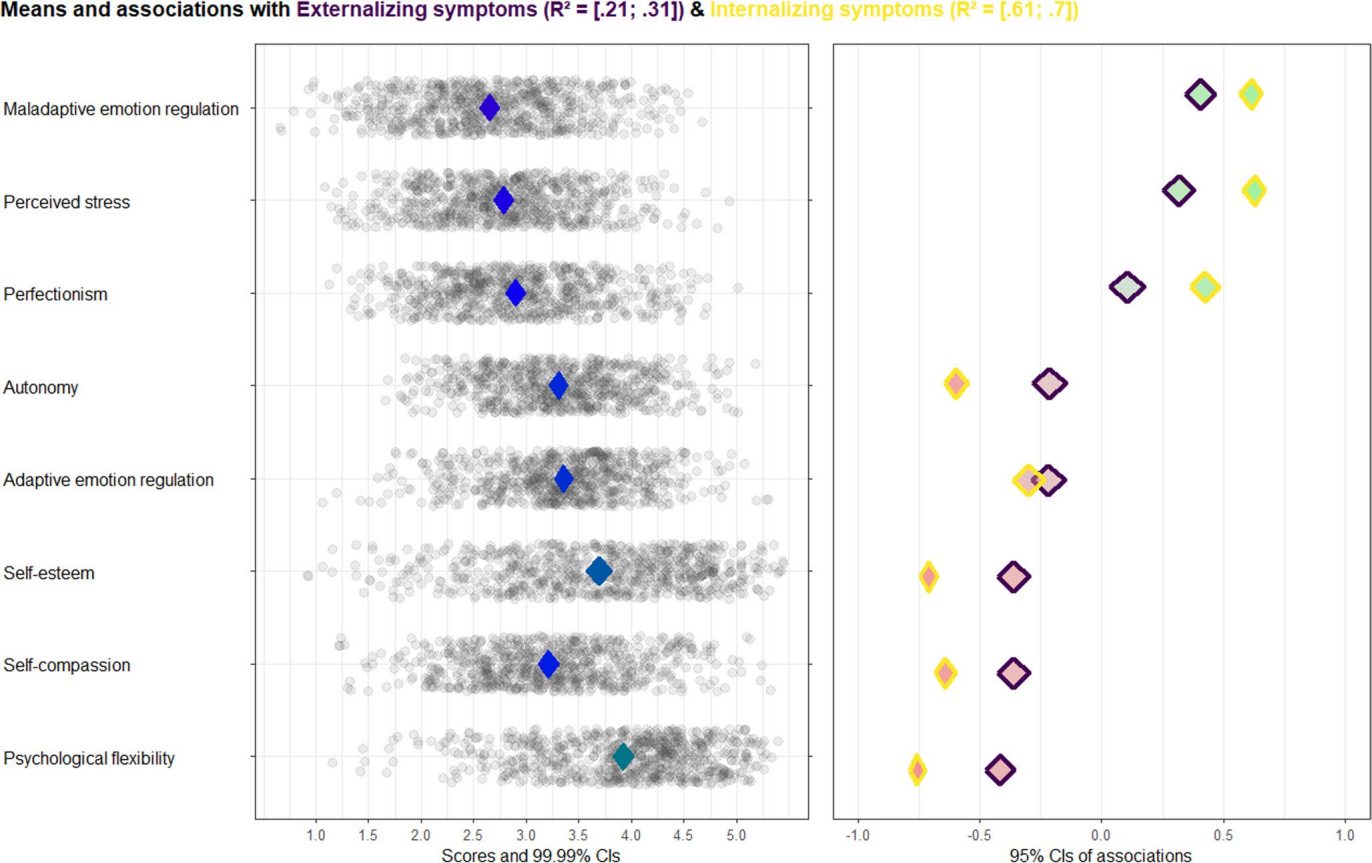
CIBER Plot

In Fig. 1, the output of the CIBER analysis is presented. The confidence intervals of the explained variance of externalizing and internalizing symptoms are presented on top of the figure. For each transdiagnostic factor, the diamonds in left-hand panel show the point estimate and 99.99% confidence intervals of the mean, ranging from 1 to 5 (i.e., representing the distribution of the transdiagnostic factors across TAY). The means varied from 2.65 (*SD* = 0.67) for maladaptive emotion regulation to 3.92 (*SD* = 0.71) for psychological flexibility. Except for psychological flexibility, the diamonds of all transdiagnostic factors are blue, indicating that the means were situated around the middle of the scale. The diamond of psychological flexibility is slightly green, indicating a high mean. The individual scores of the participants on each transdiagnostic factor are presented by the grey dots surrounding the diamonds. There was most variability in self-esteem scores (*SD* = 0.86) and least variability in autonomy scores (*SD* = 0.59). For each transdiagnostic factor, the diamonds in the right-hand panel show the point estimates and 95% confidence intervals of the correlation coefficients between each transdiagnostic factor with internalizing (i.e., yellow outline) and externalizing symptoms (i.e., purple outline; i.e., representing the strength of the associations between the transdiagnostic factors with internalizing and externalizing symptoms). The diamonds of maladaptive emotion regulation, perceived stress and perfectionism are green, representing significant positive associations between these factors and internalizing and externalizing symptoms. The diamonds of autonomy, adaptive emotion regulation, self-esteem, self-compassion and psychological flexibility are red, indicating significant negative associations between these factors and internalizing and externalizing symptoms. The diamonds with the yellow outlines (i.e., correlations with internalizing symptoms) have more intense shades than the diamonds with the purple outline (i.e., correlations with externalizing symptoms), implying that all transdiagnostic factors were more strongly related to internalizing symptoms than to externalizing symptoms (i.e., except for adaptive emotion regulation showing overlapping confidence intervals). Psychological flexibility had the strongest correlation with internalizing symptoms (*r* = −.76) and externalizing symptoms (*r*=-.42). According to the criteria of Cohen (1988), the associations between internalizing symptoms with maladaptive emotion regulation, stress, autonomy, self-esteem, self-compassion and psychological flexibility were large (i.e., *r*’s > 0.50) and the associations between internalizing symptoms with perfectionism and adaptive emotion regulation were medium (i.e., *r*’s > 0.30 and < 0.50). Moreover, the associations between externalizing symptoms with maladaptive emotion regulation, stress, self-esteem, self-compassion and psychological flexibility were medium (i.e., *r*’s > 0.30 and < 0.50) and the associations between externalizing symptoms with perfectionism, autonomy and adaptive emotion regulation were small (i.e., *r*’s > 0.10 and < 0.30).

### Latent profile analysis

#### Identifying the most optimal solution

**Fig. 2:**
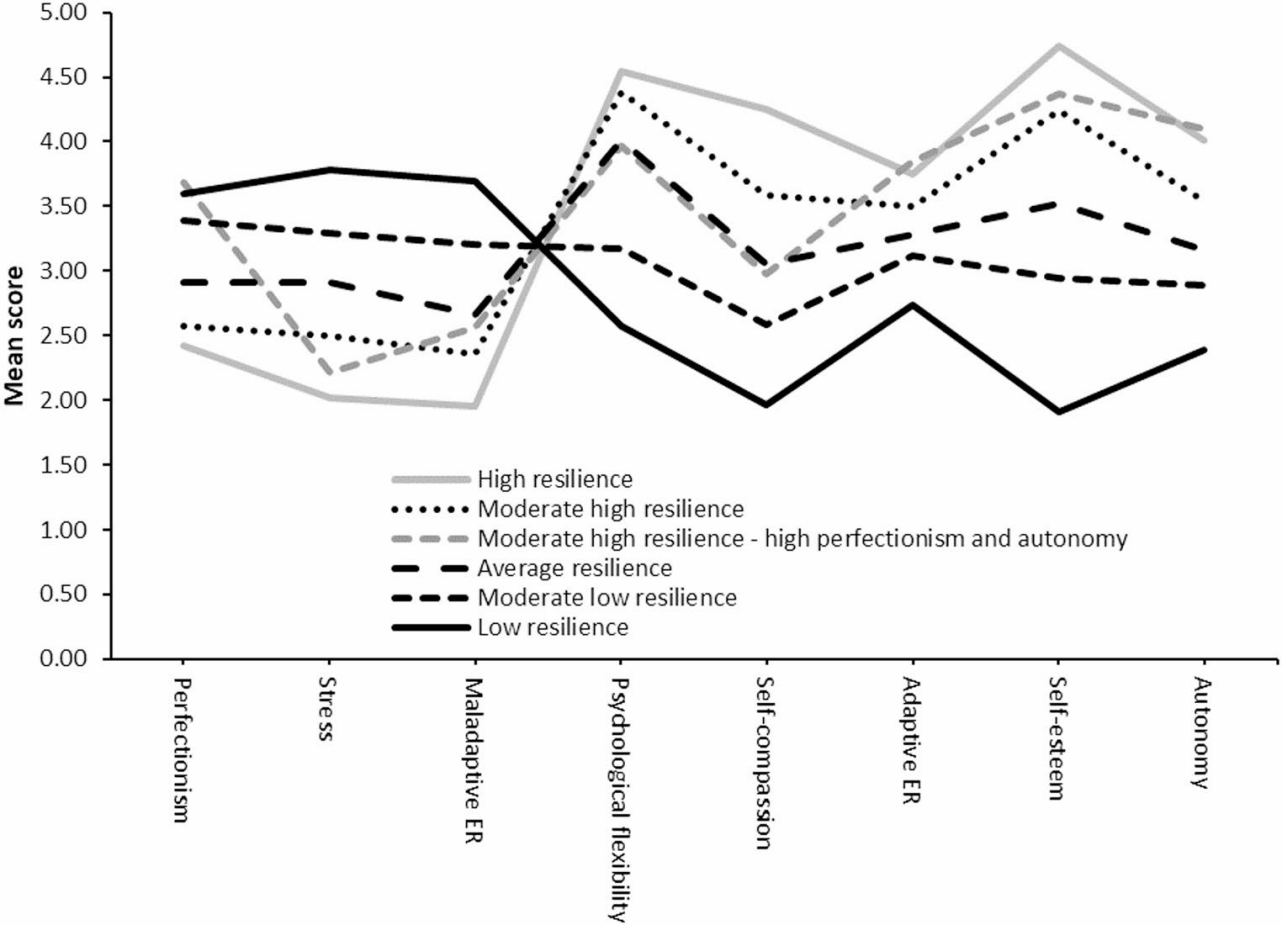
Mean Scores of the Transdiagnostic Factors for the Six Profile Solution. Note. ER = Emotion regulation

**Fig. 3:**
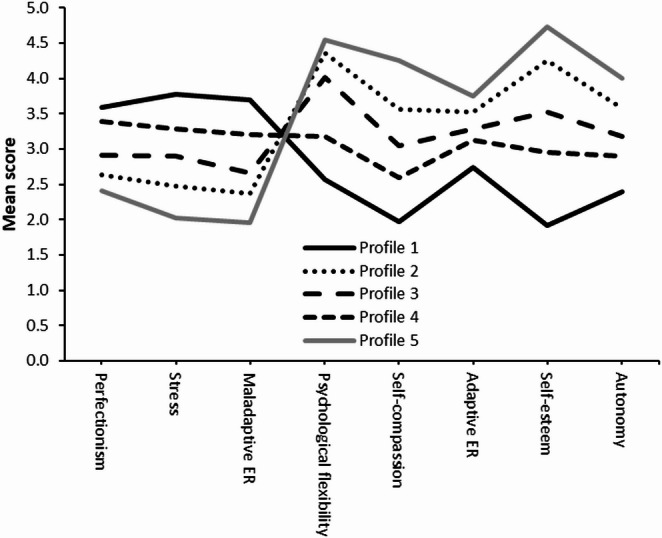
Mean Scores of the Transdiagnostic Factors for the Five Profile Solution. Note. ER = Emotion regulation

**Fig. 4:**
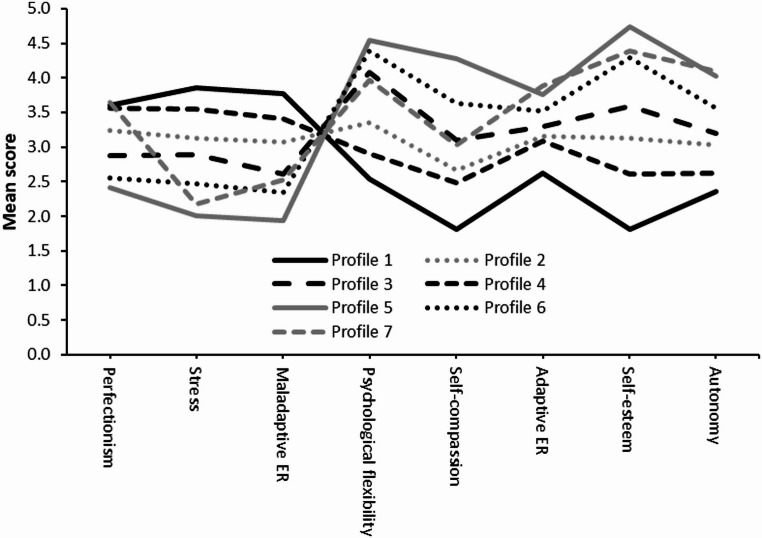
Mean Scores of the Transdiagnostic Factors for the Seven Profile Solution. Note. ER = Emotion regulation

The fit criteria for the one to seven profile solutions are presented in Table 2. As the BIC and BLRt values are considered the most robust parameters of LPA model fit, these values were prioritized (Nylund et al., 2007). The BIC suggested that the five and six profile solutions fitted the data best, as differences below the threshold of 10 indicate comparable model fit (Raftery, 1995). The BLRt indicated that the six profile solution was superior to the five profile solution and the seven profile solution fitted better than the six profile solution. Comparisons of the five, six and seven profile solutions on other fit indices (e.g., LMR, AIC, SA-BIC, and entropy) revealed minor differences. However, after inspecting the profiles (see Figs. 2 - 4), we observed that compared to the five profile solution, the six profile solution included one extra group (*n* = 13, 1.8) with a meaningful pattern, showing distinct configural differences from the other five groups (e.g., high perfectionism and autonomy with relatively average scores on other factors). The seven profile solution also identified this group but added another small group (*n* = 34, 4.6%, profile 1) with a non-meaningful pattern. Hence, considering the BIC and BLRt values, as well as the presence of an additional meaningful profile, the six profile solution was retained.

**Table 2:** Fit criteria for the one to seven profile solutions

Solution	AIC	BIC	SA-BIC	BLRt *p*	LMR *p*	Entropy *R*^2^	Mean latent profile probabilities	Smallest group size (%)
1	12019.96	12093.56	12042.75	-	-	-	1.0	735(100)
2	9840.07	9955.07	9875.68	0.000	0.000	0.887	0.96	279(38)
3	9054.97	9211.36	9103.40	0.000	0.003	0.870	0.94	178(24.2)
4	8748.25	8946.04	8809.50	0.000	0.005	0.881	0.94	53(7.2)
5	8599.18	8838.37	8673.25	0.000	0.001	0.846	0.91	49(6.7)
6	8559.59	8840.18	8646.49	0.000	0.149	0.861	0.91	13(1.8)
7	8528.43	8850.42	8628.14	0.000	0.578	0.833	0.88	12(1.6)

*AIC* Akaike information criterion, *BIC* Bayesian information criterion, *SA-BIC* Sample-size adjusted Bayesian information criterion, *BLRT* Bootstrap likelihood ratio test, *LMR* Lo–Mendell–Rubin adjusted likelihood ratio test

#### Interpreting the profile structure

To interpret the underlying group structure, we inspected the means of the transdiagnostic factors (shown in Fig. 2 and presented in Table 3). In our analysis, profile means deviating two or more standard deviations from the sample mean were considered as “extremely below/above average”, while profile means deviating one standard deviation from the sample means were considered as “below/above average”. Profile means differing half a standard deviation from the sample mean were described as “moderately below/above average”, and profile means falling within half a standard deviation from the sample mean were considered “average”. A first group of TAY (*n*=48, 6.5%) scored extremely below average on self-esteem, below average on psychological flexibility, self-compassion, adaptive emotion regulation and autonomy and above average on perfectionism, stress and maladaptive emotion regulation. This group was labelled as the “low resilience” group. A second group of TAY (*n* = 151, 20.5%) scored below average on psychological flexibility, moderately below average on self-compassion, self-esteem and autonomy, average on adaptive emotion regulation and moderately above average on perfectionism, stress and maladaptive emotion regulation. This group was labelled as the “moderate low resilience” group. A third group of TAY (*n* = 215, 29.3%) scored average on all transdiagnostic factors. This group was labelled as the “average resilience” group. A fourth group of TAY (*n* = 197, 26.8%) scored moderately below average on perfectionism, average on stress, maladaptive emotion regulation, adaptive emotion regulation and autonomy and moderately above average on psychological flexibility, self-compassion and self-esteem. This group was labelled as the “moderate high resilience” group. A fifth group of TAY (*n* = 13, 1,8%) scored moderately below average on stress, average on maladaptive emotion regulation, psychological flexibility and self-compassion, moderately above average on adaptive emotion regulation and self-esteem and above average on perfectionism and autonomy. This group was labelled as the “moderate high resilience – high perfectionism and autonomy” group. A last group of TAY (*n* = 111, 15.1%) scored below average on stress and maladaptive emotion regulation, moderately below average on perfectionism, moderately above average on psychological flexibility and adaptive emotion regulation and above average on self-compassion, self-esteem and autonomy. This group was labelled as the “high resilience” group.

**Table 3:** Means and standards errors of the transdiagnostic factors for all six profiles

Group	Perfectionism		Stress		Maladaptive ER		Psychological flexibility		Self-compassion		Adaptive ER		Self-esteem		Autonomy		
	*M*	*SE*	*M*	*SE*	*M*	*SE*	*M*	*SE*	*M*	*SE*	*M*	*SE*	*M*	*SE*	*M*	*SE*	
HR	2.42	0.05	2.02	0.06	1.96	0.06	4.54	0.03	4.25	0.07	3.75	0.05	4.74	0.04	4.01	0.06	
MHR	2.57	0.05	2.50	0.05	2.36	0.05	4.38	0.03	3.59	0.07	3.50	0.05	4.24	0.07	3.54	0.05	
MHR-HPA	3.68	0.17	2.21	0.14	2.57	0.22	3.96	0.19	2.98	0.16	3.85	0.20	4.37	0.18	4.10	0.20	
AR	2.91	0.05	2.91	0.04	2.66	0.05	4.01	0.05	3.06	0.04	3.28	0.04	3.53	0.06	3.17	0.04	
MLR	3.39	0.06	3.29	0.05	3.21	0.05	3.17	0.06	2.59	0.03	3.12	0.05	2.95	0.06	2.89	0.04	
LR	3.59	0.10	3.78	0.07	3.70	0.07	2.57	0.10	1.96	0.10	2.74	0.14	1.91	0.09	2.39	0.06	

*HR* High resilience, *MHR* Moderate high resilience, *MHR-HPA* Moderate high resilience – high perfectionism and autonomy, *AR* Average resilience, *MLR* Moderate low resilience, *LR* Low resilience

#### Correlates of group membership

To investigate group differences in internalizing symptoms and externalizing symptoms and sample (i.e., clinical or community sample), a multinomial logistic regression was performed (see Table 4). First, regarding internalizing and externalizing symptoms, we found that TAY in the lower resilience groups showed more internalizing and externalizing symptoms compared to TAY in the higher resilience groups. Overall, the order regarding the level of internalizing and externalizing symptoms was as follows: low resilience group > moderate low resilience group > average resilience group > moderate high resilience group = moderate high resilience – high perfectionism and autonomy group > high resilience group. Nonetheless, there were no significant differences in both internalizing and externalizing symptoms between TAY in the moderate high resilience – high perfectionism and autonomy group and TAY in de average resilience and moderate high resilience groups. Moreover, we did not find a significant difference in externalizing symptoms between TAY in average resilience group and TAY in the moderate high resilience group and between TAY in the moderate low resilience group and TAY in the moderate high resilience – high perfectionism and autonomy group. Second, with regard to the type of sample, we found that TAY in the lower resilience groups were more likely to be in the clinical sample than the community sample compared to TAY in the higher resilience groups. TAY in the moderate high resilience – high perfectionism and autonomy group were more likely to be in the community sample than the clinical sample, compared to all other groups. In general, the order regarding the odds of being in the clinical sample was as follows: low resilience group > moderate low resilience group > average resilience group > moderate high resilience group = high resilience group > moderate high resilience – high perfectionism and autonomy group. However, TAY in the average and moderate high resilience groups were not significantly more likely to be in the clinical sample compared to TAY in the high resilience group.

**Table 4:** Multinomial logistic regression with class membership for the six profile solution regressed on Sample, internalizing symptoms and externalizing symptoms

	Clinical Sample				Internalizing symptoms				Externalizing symptoms			
	*B*	*SE B*	*p*	OR (95% CI)	*B*	*SE B*	*p*	OR (95% CI)	*B*	*SE B*	*p*	OR (95% CI)
LR vs. MLR	−2.05	0.37	**0.000**	0.13(0.06–0.27)	−5.78	0.94	**0.000**	0.00(0.00-0.02)	−2.21	0.65	**0.001**	0.11(0.03–0.39)
LR vs. AR	−3.15	0.35	**0.000**	0.04(0.02–0.09)	−11.95	1.23	**0.000**	0.00(0.00–0.00)	−4.82	0.81	**0.000**	0.01(0.00-0.04)
LR vs. MHR-HPA	−19.70	0.90	**0.000**	0.00(0.00–0.00)	−12.37	2.84	**0.000**	0.00(0.00–0.00)	−4.14	1.15	**0.000**	0.02(0.00-0.15)
LR vs. MHR	−4.53	0.70	**0.000**	0.01(0.00-0.04)	−15.77	1.39	**0.000**	0.00(0.00–0.00)	−5.70	0.93	**0.000**	0.00(0.00-0.02)
LR vs. HR	−5.57	1.32	**0.000**	0.00(0.00-0.05)	−21.55	1.71	**0.000**	0.00(0.00–0.00)	−9.32	1.60	**0.000**	0.00(0.00–0.00)
MLR vs. AR	−1.10	0.31	**0.000**	0.33(0.18–0.62)	−6.17	0.73	**0.000**	0.00(0.00-0.01)	−2.62	0.69	**0.000**	0.07(0.02–0.28)
MLR vs. MHR-HPA	−17.66	0.87	**0.000**	0.00(0.00–0.00)	−6.59	2.66	**0.013**	0.00(0.00-0.25)	−1.94	1.06	0.068	0.14(0.02–1.15)
MLR vs. MHR	−2.48	0.64	**0.000**	0.08(0.02–0.29)	−9.99	0.80	**0.000**	0.00(0.00–0.00)	−3.49	0.79	**0.000**	0.03(0.01–0.14)
MLR vs. HR	−3.52	1.27	**0.006**	0.03(0.00-0.36)	−15.77	1.40	**0.000**	0.00(0.00–0.00)	−7.12	1.52	**0.000**	0.00(0.00-0.02)
AR vs. MHR-HPA	−16.56	0.92	**0.000**	0.00(0.00–0.00)	−0.42	2.68	0.870	0.66(0.00-100.64)	0.68	1.15	0.555	1.97(0.21–18.84)
AR vs. MHR	−1.39	0.69	**0.044**	0.25(0.07–0.96)	−3.82	0.75	**0.000**	0.02(0.01–0.10)	−0.87	0.94	0.352	0.42(0.07–2.63)
AR vs. HR	−2.43	1.26	0.054	0.09(0.01–1.05)	−9.60	1.21	**0.000**	0.00(0.00–0.00)	−4.50	1.48	**0.002**	0.01(0.00-0.20)
MHR-HPA vs. MHR	15.17	1.04	**0.000**	-	−3.40	2.64	0.198	0.03(0.00-5.91)	−1.55	1.24	0.210	0.21(0.02–2.41)
MHR-HPA vs. HR	14.13	1.51	**0.000**	-	−9.18	2.81	**0.001**	0.00(0.00-0.03)	−5.18	1.76	**0.003**	0.01(0.00-0.18)
MHR vs. HR	−1.04	1.50	0.487	0.35(0.02–6.65)	−5.78	1.18	**0.000**	0.00(0.00-0.03)	−3.63	1.71	**0.034**	0.03(0.00-0.76)

*OR* Odds Ratio, *CI* Confidence Interval, *SE* Standard Error, *LR* Low resilience, *MLR* Moderate low resilience, *AR* Average resilience, *MHR-HPA* Moderate high resilience – high perfectionism and autonomy, *MHR* Moderate high resilience, *HR* High resilience

## Discussion

The current study investigated which transdiagnostic factors (i.e., perfectionism, perceived stress, self-compassion, psychological flexibility, adaptive and maladaptive emotion regulation, self-esteem and autonomy) are the most relevant factors that are related to internalizing and externalizing symptoms in TAY. Based on CIBER, no specific transdiagnostic factors were identified as most relevant for internalizing and externalizing symptoms. All transdiagnostic factors were associated with both types of symptoms, with almost all factors being more strongly associated with internalizing symptoms than with externalizing symptoms. While psychological flexibility showed the strongest association with both types of symptoms, it was also the factor with the highest mean score (i.e., the means of all other transdiagnostic factors were situated around the middle of the scale). We only found that, compared to the other factors, perfectionism and adaptive emotion regulation had slightly weaker associations with internalizing symptoms and perfectionism, adaptive emotion regulation and autonomy were somewhat less strongly related to externalizing symptoms. Using LPA, six groups of TAY were identified: low resilience (6.5%), moderate low resilience (20.5%), average resilience (29.3%), moderate high resilience (26.8%), moderate high resilience – high perfectionism and autonomy (1.8%) and high resilience (15.1%). In general, TAY in the lower resilience groups were more likely to be in the clinical sample compared to the community sample and showed more internalizing and externalizing symptoms compared to the higher resilience groups. Conversely, TAY in the moderate high resilience – high perfectionism and autonomy group were most likely to be in the community sample compared to the clinical sample.

As, to our knowledge, the current study was the first study using the CIBER approach to establish the most relevant transdiagnostic factors of internalizing and externalizing symptoms in TAY, no hypotheses were formulated. Our results suggest that all eight factors are relevant for both internalizing and externalizing symptoms in TAY. This is in line with studies showing that individuals with higher levels of perfectionism (Limburg et al., 2017) and perceived stress (Felton et al., 2017; Thorsen et al., 2016) and lower levels of self-compassion (MacBeth & Gumley, 2012; Zessin et al., 2015), psychological flexibility (Doorley et al., 2020), emotion regulation (Aldao et al., 2010), self-esteem (Zeigler-Hill, 2011) and autonomy (Ryan et al., 2016) are more vulnerable to experience psychological problems than individuals with lower levels of perfectionism and perceived stress and higher levels of self-compassion, psychological flexibility, emotion regulation, self-esteem and autonomy. Our results also suggest that almost all transdiagnostic factors are more relevant for internalizing symptoms than for externalizing symptoms. This is in line with Aldao et al. (2010), Whitney et al. (2022) and Inguglia et al. (2015) who also found that emotion regulation, perceived stress and autonomy were more strongly related to internalizing symptoms than to externalizing symptoms. A potential explanation for this finding is that the eight transdiagnostic factors are primarily associated with internal processes (e.g., negative self-judgement, rumination, fear of failure) which play an important role in internalizing symptoms. Conversely, externalizing symptoms are predominantly behaviorally oriented, suggesting a weaker and more indirect association with such internal processes and the eight transdiagnostic factors. However, differences in association strengths could also be explained by the relatively low levels of externalizing symptoms in our sample (i.e., the level of externalizing symptoms was significantly lower than the level of internalizing symptoms).

Furthermore, the CIBER plot revealed that psychological flexibility had the strongest correlation with both internalizing and externalizing symptoms. This indicates that psychological flexibility might be an important intervention target for clinically referred TAY with problems in this area (i.e., especially as descriptive analyses showed that TAY from the clinical sample exhibited lower levels of psychological flexibility compared to those in the community sample). However, given that most TAY, particularly in the community sample, already demonstrate relatively high scores in this area, it raises the question whether psychological flexibility is the most relevant factor to address in prevention programs. Moreover, since the means of all other transdiagnostic factors were situated around the middle of the scale, we could not identify the most relevant transdiagnostic factor(s) based on their distribution across TAY. We only found that perfectionism and adaptive emotion regulation showed somewhat weaker associations with both internalizing and externalizing symptoms, and autonomy exhibited a slightly weaker associations with externalizing symptoms. Regarding emotion regulation, this finding is in line with the meta-analysis of Aldao et al. (2010) stating that the presence of maladaptive emotion regulation strategies might be more disadvantageous than the relative lack of adaptive emotion regulation strategies. This suggests that maladaptive emotion regulation might be more relevant to target in interventions for TAY compared to adaptive emotion regulation. Moreover, according to the two-factor model of perfectionism, perfectionism consists of two higher-order dimensions: perfectionistic strivings (e.g., high personal standards and striving for perfection) and perfectionistic concerns (e.g., concern over mistakes or fear of other’s negative evaluations) (Stoeber et al., 2020). Perfectionistic concerns are often maladaptive, whereas perfectionistic strivings can be adaptive. Hence, perfectionism might not be a univariate construct that is inherently related to negative outcomes and therefore as a univariate construct not that predictive for psychological symptoms. This proposes that in interventions for TAY, specifically perfectionistic concerns should be targeted. Furthermore, as mentioned earlier, these results also suggest that autonomy might be a more relevant intervention target for TAY with internalizing symptoms than for TAY with externalizing symptoms (Inguglia et al., 2015). Notwithstanding, it should be noted that differences in correlation strengths were not very profound and more research is needed to further support our suggestions regarding interventions and prevention targets.

The present study was also the first executing an LPA on the abovementioned eight transdiagnostic factors. In line with our hypothesis, we found six groups that differed in their general levels of several transdiagnostic factors, but also could be labeled as more or less resilient based on their associations with the three mental health related outcomes (e.g., internalizing symptoms, externalizing symptoms and type of sample [clinical or community sample]). Although we considered the possibility of identifying groups of TAY that predominantly encounter challenges in specific areas (e.g., TAY that struggle primarily with self-related issues represented by low self-compassion and self-esteem), we did not identify certain groups. Our results showed that all transdiagnostic factors are highly related to each other. Specifically, TAY who experience difficulties regarding one of the transdiagnostic factors (i.e., high scores on perfectionism, stress, maladaptive emotion regulation or low scores self-compassion, psychological flexibility, autonomy, adaptive emotion regulation) are also likely to have difficulties with the other transdiagnostic factors and experience more internalizing and externalizing symptoms. An exploratory factor analysis confirmed this finding. Explicitly, we identified one factor underlying all eight transdiagnostic factors. This factor could explain more than half of the variance in the eight transdiagnostic factors and was negatively related to internalizing and externalizing symptoms. Essentially, this suggests that the various transdiagnostic factors were closely interconnected, forming a key underlying factor that was related to psychological symptoms. Interestingly, also here, adaptive emotion regulation, perfectionism and autonomy had the lowest eigenvalues which indicate that these variables contribute least to explaining the underlying structure of the data.

Although all transdiagnostic factors were highly related to each other, there was one exception. Specifically, we found one group of TAY who had relatively average scores on all transdiagnostic factors, but extremely high levels of perfectionism and autonomy (i.e., TAY in moderate high resilience – high perfectionism and autonomy group). Despite their high levels of perfectionism, these TAY showed less internalizing and externalizing symptoms compared to TAY in the low resilience and moderate low resilience groups and equal symptoms compared to TAY in the average and moderate high resilience group. Moreover, TAY in this group were more likely to be in the community sample than in de clinical sample. This finding supports the two-factor model of perfectionism and indicates that perfectionism might be not necessarily maladaptive (Stoeber et al., 2020).

The study had some limitations that should be mentioned. In general, although we included a clinical sample of TAY, the level of psychological symptoms in our sample were low, especially regarding externalizing symptoms. Moreover, most participants were highly educated Dutch females. To enhance the generalizability of the findings, future studies could investigate these transdiagnostic factors in larger and more diverse samples of clinically referred TAY. In addition, we solely relied on self-report data which raises the issue of common method variance. This issue might have contributed to the high correlations among the transdiagnostic factors as well as between these factors and both internalizing and externalizing symptoms. Future studies could build on our findings by including multiple informants (e.g., parents, clinicians, peers) or other measures (e.g., clinical interviews or observations). Furthermore, the data was cross-sectional. Hence, we could not make any conclusions regarding the direction and temporal order of the associations between the eight transdiagnostic factors and internalizing and externalizing symptoms nor make causal claims. Moreover, we were unable to examine the stability or possible change in the profiles over time. However, it still offers valuable preliminary insights to guide future research and intervention development.

Notwithstanding these limitations, the study had some important strengths. To our knowledge the current study was the first investigating which transdiagnostic factors are the most relevant factors that are related to internalizing and externalizing symptoms in clinical and non-clinical TAY using both a variable-centred and person-centred approach. Employing both approaches is beneficial as they offer complementary perspectives on data analysis, capturing both general patterns and individual differences within TAY. Moreover, we selected eight psychological transdiagnostic factors based on theoretical ground and empirical evidence (e.g., Limburg et al., 2017). Focusing on psychological transdiagnostic factors is relevant as it allows researchers and clinicians to identify common underlying mechanism that are related to various types of psychological symptoms, transcending traditional diagnostic boundaries.

To conclude, all eight transdiagnostic factors (i.e., perfectionism, perceived stress, self-compassion, psychological flexibility, adaptive and maladaptive emotion regulation, self-esteem and autonomy) seem relevant for both internalizing and externalizing symptoms in TAY. This underscores the importance for further research on transdiagnostic factors and transdiagnostic interventions for TAY, particularly given the comorbid problems and shifting symptom profiles often observed in this age group (Kessler et al., 2005; Marchette & Weisz, 2017). Moreover, almost all factors were more strongly related to internalizing symptoms compared to externalizing symptoms. This implies that focusing on these eight transdiagnostic factors for TAY with internalizing problems is particularly relevant. However, this finding might also be attributed to the low levels of externalizing symptoms in our sample. Last, although psychological flexibility showed the strongest association with internalizing and externalizing symptoms, it was also the factor where most TAY had high scores on. Moreover, while adaptive emotion regulation, perfectionism and autonomy seemed somewhat less strongly related to internalizing and/or externalizing symptoms compared to the other factors, no profound differences were found. Hence, based on our analyses, all transdiagnostic factors seemed equally relevant. Therefore, as a next step, future research could investigate which of the transdiagnostic factors are most malleable to change. Although there are already studies showing that interventions can improve some of our transdiagnostic factors in TAY (e.g., emotion regulation and stress) (Moltrecht et al., 2021; Van Loon et al., 2020), there is a lack of studies explicitly comparing the changeability across our eight transdiagnostic factors. The factors that appear most easy to change are potentially the most relevant factors to target in prevention and intervention programs for TAY. Especially as, given the high correlations among the transdiagnostic factors, changing one transdiagnostic factor might not only be related to change in psychological symptoms, but also in the other transdiagnostic factors. Notwithstanding, prevention and intervention studies are needed to confirm this.

## Data Availability

As the data contains sensitive data (e.g., potential patient identifiers) the data that support the findings of this study are not openly available. Data are located in controlled access data storage at Utrecht University. Permission to access the data can be provided by Denise Bodden (d.bodden@uu.nl) upon reasonable request.

## Appendix

An exploratory factor analysis was conducted using principal axis factoring with promax rotation. One factor (with an eigenvalue exceeding 1) was identified as underlying the eight transdiagnostic factors. This factor accounted for around 55.4% of the variance in the eight transdiagnostic factors. In ascending order, the factor loading were as follows: adaptive emotion regulation (.41), perfectionism (-.56), autonomy (.73), maladaptive emotion regulation (-.74), perceived stress (-.78), psychological flexibility (.81), self-esteem (.88) and self-compassion (.90). Additionally, the factor showed a moderate negative correlation with externalizing symptoms (*r*=-0.41, *p*<.001) and a strong negative correlation with internalizing symptoms (*r*=-0.77, *p* <.001). TAY in the clinical sample scored significantly lower on this factor compared to TAY in the community sample (*t*=12.23, *p* <.001)

## Footnotes

1 The clinical sample differed from the community sample in demographics and scores on transdiagnostic factors, internalizing symptoms, and externalizing symptoms. Consequently, we conducted the CIBER and LPA analyses twice. For the CIBER analysis, we examined the clinical, community, and combined samples separately. We observed highly similar graphs and could draw consistent conclusions across all samples. Regarding the LPA, we included the sample type (0 = community sample and 1 = clinical sample) as a covariate and found only marginal differences compared to analyses without it, resulting in similar conclusions. Therefore, to increase statistical power, we opted to run the primary analyses without including the sample type as a covariate.

## Declarations

### Pre-registration

We preregistered the study in Open Science Framework: https://osf.io/59fpk/?view_only=f5212a02b58b4354a1b75cf3c4a0c0b7

## References

[CR1] Aldao, A., Nolen-Hoeksema, S., & Schweizer, S. (2010). Emotion-regulation strategies across psychopathology: A meta-analytic review. *Clinical Psychology Review*, *30*(2), 217–237. 10.1016/j.cpr.2009.11.004 20015584 10.1016/j.cpr.2009.11.004

[CR2] American Psychiatric Association (2013). *Diagnostic and statistical manual of mental disorders: DSM-5*.

[CR3] Asparouhov, T., & Muthén, B. (2014). Auxiliary variables in mixture modeling: Three-step approaches using M plus. *Structural Equation Modeling: A Multidisciplinary Journal,**21*(3), 329–341. 10.1080/10705511.2014.915181

[CR4] Beveren, M., Braet, C., Cracco, E., Theuwis, L., Grob, A., & Smolenski, C. (2020). *FEEL-KJ: Vragenlijst over emotieregulatie bij kinderen en jongeren*. Hogrefe.

[CR5] Bi, D., & Li, X. (2021). Psychological flexibility profiles, college adjustment, and subjective well-being among college students in China: A latent profile analysis. *Journal of Contextual Behavioral Science,**20*, 20–26. 10.1016/j.jcbs.2021.01.008

[CR6] Bollen, K. A., & Long, J. S. (1993). *Testing Structural Equation Models*. Sage.

[CR7] Brown, J., & Marshall, M. (2006). The thee faces of self-esteem. In W. Kernis (Ed.), *Self-esteem issues and answers: A source book of current perspectives* (pp. 4–9). Psychology Press.

[CR8] Celeux, G., & Soromenho, G. (1996). An entropy criterion for assessing the number of clusters in a mixture model. *Journal of Classification,**13*, 195–212. 10.1007/BF01246098

[CR9] Chi, X., Huang, L., Zhang, J., Wang, E., & Ren, Y. (2022). Latent profiles of multi-dimensionality of self-compassion predict youth psychological adjustment outcomes during the COVID-19: A longitudinal mixture regression analysis. *Current Psychology*, *43*(14), 13190–13201. 10.1007/s12144-022-03378-3 10.1007/s12144-022-03378-3 PMC9273687 35846239

[CR10] Cohen, J. (1988). *Statistical power analysis for the behavioral sciences*. Lawrence Erlbaum Associates.

[CR11] Cohen, S., Kamarck, T., & Mermelstein, R. (1983). A global measure of perceived stress. *Journal of Health and Social Behavior*, *24*, 385–396. 6668417

[CR12] Collins, L. M., & Lanza, S. T. (2010). *Latent class and latent transition analysis with applications in the social, behavioral and health sciences*. Wiley.

[CR13] Costello, E. J., & Maughan, B. (2015). Annual research review: Optimal outcomes of child and adolescent mental illness. *Journal of Child Psychology and Psychiatry*, *56*(3), 324–341. 10.1111/jcpp.12371 25496295 10.1111/jcpp.12371 PMC4557213

[CR14] Cracco, E., Van Durme, K., & Braet, C. (2015). Validation of the FEEL-KJ: An instrument to measure emotion regulation strategies in children and adolescents. *PloS One,**10*(9), e0137080. 10.1371/journal.pone.0137080 26331845 10.1371/journal.pone.0137080 PMC4557990

[CR15] Craig, C., Hiskey, S., & Spector, A. (2020). Compassion focused therapy: A systematic review of its effectiveness and acceptability in clinical populations. *Expert Review of Neurotherapeutics*, *20*(4), 385–400. 10.1080/14737175.2020.1746184 32196399 10.1080/14737175.2020.1746184

[CR16] Cronbach, L. J. (1951). Coefficient alpha and the internal structure of tests. *Psychometrika*, *16*(3), 297–334. 10.1007/bf02310555

[CR17] Crutzen, R., Peters, G. J. Y., & Noijen, J. (2017). Using confidence interval-based estimation of relevance to select social-cognitive determinants for behavior change interventions. *Frontiers in Public Health,**5*, 165. 10.3389/fpubh.2017.00165 28785553 10.3389/fpubh.2017.00165 PMC5508122

[CR18] Dixon-Gordon, K. L., Aldao, A., & De Los Reyes, A. (2015). Repertoires of emotion regulation: A person-centered approach to assessing emotion regulation strategies and links to psychopathology. *Cognition and Emotion,**29*(7), 1314–1325. 10.1080/02699931.2014.983046 25435338 10.1080/02699931.2014.983046

[CR19] Doorley, J. D., Goodman, F. R., Kelso, K. C., & Kashdan, T. B. (2020). Psychological flexibility: What we know, what we do not know, and what we think we know. *Social and Personality Psychology Compass*, *14*(12), 1–11. 10.1111/spc3.12566

[CR20] Felton, J. W., Banducci, A. N., Shadur, J. M., Stadnik, R., MacPherson, L., & Lejuez, C. W. (2017). The developmental trajectory of perceived stress mediates the relations between distress tolerance and internalizing symptoms among youth. *Development and Psychopathology,**29*(4), 1391–1401. 10.1017/S0954579417000335 28318473 10.1017/S0954579417000335 PMC6360527

[CR21] Fergus, T. A., Valentiner, D. P., Gillen, M. J., Hiraoka, R., Twohig, M. P., Abramowitz, J. S., & McGrath, P. B. (2012). Assessing psychological inflexibility: The psychometric properties of the avoidance and fusion questionnaire for youth in two adult samples. *Psychological Assessment,**24*(2), 402–408. 10.1037/a0025776 21988185 10.1037/a0025776

[CR22] Ferrari, M., Yap, K., Scott, N., Einstein, D. A., & Ciarrochi, J. (2018). Self-compassion moderates the perfectionism and depression link in both adolescence and adulthood. *PloS One*, *13*(2), e0192022. 10.1371/journal.pone.0192022 29466452 10.1371/journal.pone.0192022 PMC5821438

[CR23] Ferrari, M., Beath, A., Einstein, D. A., Yap, K., & Hunt, C. (2022). Gender differences in self-compassion: A latent profile analysis of compassionate and uncompassionate self-relating in a large adolescent sample. *Current Psychology,**42*(28), 24132–24147. 10.1007/s12144-022-03408-0

[CR24] Franck, E., De Raedt, R., Barbez, C., & Rosseel, Y. (2008). Psychometric properties of the Dutch Rosenberg self-esteem scale. *Psychologica Belgica*, *48*(1), 25–35. 10.5334/pb-48-1-25

[CR25] Frost, R. O., Marten, P., Lahart, C., & Rosenblate, R. (1990). The dimensions of perfectionism. *Cognitive Therapy and Research*, *14*, 449–468. 10.1007/BF01172967

[CR26] Greco, L. A., Lambert, W., & Baer, R. A. (2008). Psychological inflexibility in childhood and adolescence: Development and evaluation of the avoidance and fusion questionnaire for youth. *Psychological Assessment,**20*(2), 93–102. 10.1037/1040-3590.20.2.93 18557686 10.1037/1040-3590.20.2.93

[CR27] Gustavson, K., Knudsen, A. K., Nesvåg, R., Knudsen, G. P., Vollset, S. E., & Reichborn-Kjennerud, T. (2018). Prevalence and stability of mental disorders among young adults: Findings from a longitudinal study. *BMC Psychiatry,**18*, 1–15. 10.1186/s12888-018-1647-5 29530018 10.1186/s12888-018-1647-5 PMC5848432

[CR28] Hayes, S. C., Luoma, J. B., Bond, F. W., Masuda, A., & Lillis, J. (2006). Acceptance and commitment therapy: Model, processes and outcomes. *Behaviour Research and Therapy,**44*(1), 1–25. 10.1016/j.brat.2005.06.006 16300724 10.1016/j.brat.2005.06.006

[CR29] IBM (2020). *Transforming different Likert scales to a common scale*. Retrieved April 16 from https://www.ibm.com/support/pages/transforming-different-likert-scales-common-scale

[CR30] Inguglia, C., Ingoglia, S., Liga, F., Lo Coco, A., & Lo Cricchio, M. G. (2015). Autonomy and relatedness in adolescence and emerging adulthood: Relationships with parental support and psychological distress. *Journal of Adult Development*, *22*(1), 1–13. 10.1007/s10804-014-9196-8

[CR31] Keulen, J., Matthijssen, D., Schraven, J., Deković, M., & Bodden, D. (2023). The effectiveness and cost-effectiveness of Acceptance and Commitment Therapy as a transdiagnostic intervention for transitional-age youth: study protocol of a randomized controlled trial. *BMC Psychiatry,**23*(51), 1–16. 10.1186/s12888-023-04535-z 36658510 10.1186/s12888-023-04535-z PMC9850708

[CR32] Kessler, R. C., Berglund, P., Demler, O., Jin, R., Merikangas, K. R., & Walters, E. E. (2005). Lifetime prevalence and age-of-onset distributions of DSM-IV disorders in the National Comorbidity Survey Replication. *Archives of General Psychiatry,**62*(6), 593–602. 10.1001/archpsyc.62.6.593 15939837 10.1001/archpsyc.62.6.593

[CR33] Korrelboom, K., IJdema, T., Karreman, A., & van der Gaag, M. (2022). The effectiveness of transdiagnostic applications of competitive memory training (COMET) on low self-esteem and comorbid depression: A meta-analysis of randomized controlled trials. *Cognitive Therapy and Research,**46*(3), 532–543. 10.1007/s10608-021-10286-6

[CR34] Langeheine, R., Pannekoek, J., & Van de Pol, F. (1996). Bootstrapping goodness-of-fit measures in categorical data analysis. *Sociological Methods & Research,**24*(4), 492–516. 10.1177/0049124196024004004

[CR35] Lee, E. H. (2012). Review of the psychometric evidence of the perceived stress scale. *Asian Nursing Research,**6*(4), 121–127. 10.1016/j.anr.2012.08.004 25031113 10.1016/j.anr.2012.08.004

[CR36] Limburg, K., Watson, H. J., Hagger, M. S., & Egan, S. J. (2017). The relationship between perfectionism and psychopathology: A meta-analysis. *Journal of Clinical Psychology,**73*(10), 1301–1326. 10.1002/jclp.22435 28026869 10.1002/jclp.22435

[CR37] Lo, Y., Mendell, N. R., & Rubin, D. B. (2001). Testing the number of components in a normal mixture. *Biometrika*, *88*(3), 767–778. 10.1093/biomet/88.3.767

[CR38] Lynch, S. J., Sunderland, M., Newton, N. C., & Chapman, C. (2021). A systematic review of transdiagnostic risk and protective factors for general and specific psychopathology in young people. *Clinical Psychology Review*, *87*, 102036. 10.1016/j.cpr.2021.102036 33992846 10.1016/j.cpr.2021.102036

[CR39] MacBeth, A., & Gumley, A. (2012). Exploring compassion: A meta-analysis of the association between self-compassion and psychopathology. *Clinical Psychology Review*, *32*(6), 545–552. 10.1016/j.cpr.2012.06.003 22796446 10.1016/j.cpr.2012.06.003

[CR40] Marchette, L. K., & Weisz, J. R. (2017). Practitioner review: Empirical evolution of youth psychotherapy toward transdiagnostic approaches. *Journal of Child Psychology and Psychiatry*, *58*(9), 970–984. 10.1111/jcpp.12747 28548291 10.1111/jcpp.12747

[CR41] Marshall, E. J., & Brockman, R. N. (2016). The relationships between psychological flexibility, self-compassion, and emotional well-being. *Journal of Cognitive Psychotherapy*, *30*(1), 60–72. 10.1891/0889-8391.30.1.60 32755906 10.1891/0889-8391.30.1.60

[CR42] Martel, A., & Fuchs, D. C. (2017). Transitional age youth and mental illness–influences on young adult outcomes. *Child and Adolescent Psychiatric Clinics of North America,**26*(2), xiii–xvii. 10.1016/j.chc.2017.01.001 28314464 10.1016/j.chc.2017.01.001

[CR43] McGlinchey, E., Kirby, K., McElroy, E., & Murphy, J. (2021). The role of emotional regulation in anxiety and depression symptom interplay and expression among adolescent females. *Journal of Psychopathology and Behavioral Assessment*, *43*(4), 854–868. 10.1007/s10862-021-09883-2

[CR44] Moltrecht, B., Deighton, J., Patalay, P., & Edbrooke-Childs, J. (2021). Effectiveness of current psychological interventions to improve emotion regulation in youth: A meta-analysis. *European Child & Adolescent Psychiatry,**30*(6), 829–848. 10.1007/s00787-020-01498-4 32108914 10.1007/s00787-020-01498-4 PMC8140974

[CR45] Muthén, L. K., & Muthén, B. O. (2017). *Mplus user’s guide* (8th ed.). Muthén & Muthén.

[CR46] Neff, K. D. (2003a). Self-compassion: An alternative conceptualization of a healthy attitude toward oneself. *Self and Identity*, *2*(2), 85–101. 10.1080/15298860309032

[CR47] Neff, K. D. (2003b). The development and validation of a scale to measure self-compassion. *Self and Identity, 2*(3), 223–250. 10.1080/15298860309027

[CR48] Neff, K. D. (2016). The self-compassion scale is a valid and theoretically coherent measure of self-compassion. *Mindfulness*, *7*(1), 264–274. 10.1007/s12671-015-0479-3

[CR49] Noom, M. J., Deković, M., & Meeus, W. (2001). Conceptual analysis and measurement of adolescent autonomy. *Journal of Youth and Adolescence*, *30*(5), 577–595. 10.1023/A:1010400721676

[CR50] Nye, A., Delgadillo, J., & Barkham, M. (2023). Efficacy of personalized psychological interventions: A systematic review and meta-analysis. *Journal of Consulting and Clinical Psychology*, *91*(7), 389. 10.1037/ccp0000820 37166831 10.1037/ccp0000820

[CR51] Nylund, K. L., Asparouhov, T., & Muthén, B. O. (2007). Deciding on the number of classes in latent class analysis and growth mixture modeling: A Monte Carlo simulation study. *Structural Equation Modeling: A Multidisciplinary Journal*, *14*(4), 535–569. 10.1080/10705510701575396

[CR52] Patel, V., Flisher, A. J., Hetrick, S., & McGorry, P. (2007). Mental health of young people: A global public-health challenge. *The Lancet,**369*(9569), 1302–1313. 10.1016/S0140-6736(07)60368-7 10.1016/S0140-6736(07)60368-7 17434406

[CR53] Peters, G. J. Y. (2017). *Userfriendlyscience: Quantitive analysis made accessible*.

[CR54] Peters, G. J., & Crutzen, R. (2018). Establishing determinant relevance using CIBER: An introduction and tutorial. *European Health Psychologist*, *20*(3), 484–494. 10.31234/osf.io/5wjy4

[CR55] Raes, F., Pommier, E., Neff, K. D., & Van Gucht, D. (2011). Construction and factorial validation of a short form of the Self-Compassion scale. *Clinical Psychology & Psychotherapy,**18*(3), 250–255. 10.1002/cpp.702 21584907 10.1002/cpp.702

[CR56] Raftery, A. E. (1995). Bayesian model selection in social research. *Sociological Methodology,**25*, 111–163. 10.2307/271063

[CR57] Rosenberg, M. (1965). *Society and the adolescent self-image*. Princeton University Press.

[CR58] Rudolph, S. G., Flett, G. L., & Hewitt, P. L. (2007). Perfectionism and deficits in cognitive emotion regulation. *Journal of Rational-Emotive & Cognitive-Behavior Therapy*, *25*(4), 343–357. 10.1007/s10942-007-0056-3

[CR59] Ruiz-Esteban, C., Méndez, I., Fernández-Sogorb, A., & Álvarez Teruel, J. D. (2021). Perfectionism classes and aggression in adolescents. *Frontiers in Psychology*, *12*, 686380. 10.3389/fpsyg.2021.686380 34140922 10.3389/fpsyg.2021.686380 PMC8204804

[CR60] Ryan, R. M., Deci, E. L., & Vansteenkiste, M. (2016). Autonomy and autonomy disturbances in self-development and psychopathology: Research on motivation, attachment, and clinical process. In D. Cicchetti & D. J. Cohen (Eds.), *Developmental Psychopathology* (pp. 385–438).

[CR61] Sakiris, N., & Berle, D. (2019). A systematic review and meta-analysis of the unified protocol as a transdiagnostic emotion regulation based intervention. *Clinical Psychology Review*, *72*, 101751. 10.1016/j.cpr.2019.101751 31271848 10.1016/j.cpr.2019.101751

[CR62] Simon, E., & Verboon, P. (2016). Psychological inflexibility and child anxiety. *Journal of Child and Family Studies*, *25*(12), 3565–3573. 10.1007/s10826-016-0522-6 27891046 10.1007/s10826-016-0522-6 PMC5104759

[CR63] Stoeber, J., Madigan, D. J., & Gonidis, L. (2020). Perfectionism is adaptive and maladaptive, but what’s the combined effect? *Personality and Individual Differences,**161*, Article 109846. 10.1016/j.paid.2020.109846

[CR64] Stornæs, A. V., Rosenvinge, J. H., Sundgot-Borgen, J., Pettersen, G., & Friborg, O. (2019). Profiles of perfectionism among adolescents attending specialized elite-and ordinary lower secondary schools: A Norwegian cross-sectional comparative study. *Frontiers in Psychology*, *10*, 2039. 10.3389/fpsyg.2019.02039 31551878 10.3389/fpsyg.2019.02039 PMC6743349

[CR65] Thorsen, F., Antonson, C., Sundquist, J., & Sundquist, K. (2016). Perceived stress and psychiatric symptoms in Swedish upper secondary school students. *Journal of Educational and Developmental Psychology*, *6*(2), 183–194. 10.5539/jedp.v6n2p183

[CR66] Van Den Heuvel, M. W., Stikkelbroek, Y. A., Bodden, D. H., & Van Baar, A. L. (2020). Coping with stressful life events: Cognitive emotion regulation profiles and depressive symptoms in adolescents. *Development and Psychopathology*, *32*(3), 985–995. 10.1017/S0954579419000920 31370910 10.1017/S0954579419000920

[CR67] Van der Ploeg, J., & Van der Ploeg, J.D. (2013). *Stress bij kinderen*. Bohn Stafleu van Loghum.

[CR68] Van Loon, A. W., Creemers, H. E., Beumer, W. Y., Okorn, A., Vogelaar, S., Saab, N., Miers, A. C., Westenberg, P. M., & Asscher, J. J. (2020). Can schools reduce adolescent psychological stress? A multilevel meta-analysis of the effectiveness of school-based intervention programs. *Journal of Youth and Adolescence*, *49*, 1127–1145. 10.1007/s10964-020-01201-5 32034632 10.1007/s10964-020-01201-5 PMC7237523

[CR69] Verhulst, F. C., van der Ende, J., & Koot, J. M. (1997). *Youth Self-Report (YSR)*. Academic Medical Center Rotterdam/Erasmus University, Sophia Children’s Hospital, Department of Child and Adolescent Psychiatry.

[CR70] Vermunt, J. K. (2010). Latent class modeling with covariates: Two improved three-step approaches. *Political Analysis*, *18*(4), 450–469. 10.1093/pan/mpq025

[CR71] Vervoort, L., Naets, T., De Guchtenaere, A., Tanghe, A., & Braet, C. (2020). Using confidence interval-based estimation of relevance to explore bottom-up and top-down determinants of problematic eating behavior in children and adolescents with obesity from a dual pathway perspective. *Appetite,**150*, Article 104676. 10.1016/j.appet.2020.104676 32198094 10.1016/j.appet.2020.104676

[CR72] Whiteford, H. A., Degenhardt, L., Rehm, J., Baxter, A. J., Ferrari, A. J., Erskine, H. E., Charlson, F. J., Norman, R. E., Flaxman, A. D., Johns, N., Burstein, R., Murray, C. J., & Vos, T. (2013). Global burden of disease attributable to mental and substance use disorders: Findings from the global burden of disease study 2010. *Lancet*, *382*(9904), 1575–1586. 10.1016/S0140-6736(13)61611-6 23993280 10.1016/S0140-6736(13)61611-6

[CR73] Whitney, S., Luther, A. W., & Ferro, M. A. (2022). Psychometric properties of the perceived stress scale in youth with mental illness. *Journal of Child and Family Studies,**31*(10), 2801–2812. 10.1007/s10826-022-02387-x

[CR74] Wilens, T. E., & Rosenbaum, J. F. (2013). Transitional aged youth: A new frontier in child and adolescent psychiatry. *Journal of the American Academy of Child and Adolescent Psychiatry*, *52*(9), 887–890. 10.1016/j.jaac.2013.04.020 23972688 10.1016/j.jaac.2013.04.020

[CR75] Williams, G. A., & Kibowski, F. (2016). Latent class analysis and latent profile analysis. In L. A. Jason & D. S. Glenwick (Eds.), *Handbook of methodological approaches to community-based research: Qualitative, quantitative, and mixed methods* (pp. 143–151).

[CR76] World Health Organization (2019). *International Statistical Classification of Diseases and Related Health Problems* (11th ed.). https://doi.org/https://icd.who.int/

[CR77] Zeigler-Hill, V. (2011). The connections between self-esteem and psychopathology. *Journal of Contemporary Psychotherapy*, *41*(3), 157–164.

[CR78] Zessin, U., Dickhäuser, O., & Garbade, S. (2015). The relationship between self-compassion and well‐being: A meta‐analysis. *Applied Psychology: Health and Well-Being,**7*(3), 340–364. 10.1111/aphw.12051 26311196 10.1111/aphw.12051

